# Temporal Shift Length and Antecedent Occurrence Likelihood Modulate Counterfactual Conditional Comprehension: Evidence from Event-Related Potentials

**DOI:** 10.3390/brainsci13121724

**Published:** 2023-12-17

**Authors:** Lingda Kong, Yong Jiang, Yan Huang, Xiaoming Jiang

**Affiliations:** 1 Institute of Corpus Studies & Applications, Shanghai International Studies University, Shanghai 200083, China; konglingda@shisu.edu.cn (L.K.); 2021006@shisu.edu.cn (Y.J.); 2 School of Foreign Languages, East China University of Science and Technology, Shanghai 200237, China; hannahhuang2019@163.com; 3 Institute of Linguistics, Shanghai International Studies University, Shanghai 200083, China

**Keywords:** temporal shift, likelihood of occurrence, implied causal relationship, Chinese counterfactuals, N400

## Abstract

Counterfactual conditionals posit hypothetical scenarios in which antecedent events contradict reality. This study examined whether and how the processing difficulty of Chinese counterfactual conditionals (*yaobushi*, equivalent to *if it had not been for* in English) can be affected by the length of temporal shifts of the events across clauses and the likelihood of the antecedent occurrence. Participants read Chinese counterfactuals that contained either long (e.g., *qunian-xianzai* [last year-right now]) or short temporal shifts (e.g., *zuotian-xianzai* [yesterday-right now]) within highly likely (e.g., sign up for school activity) or less likely contexts (e.g., sign up for Arctic scientific research). ERP results revealed a significant N400 interaction between the temporal shift length and antecedent likelihood on the temporal indicators in the consequent and the sentence-ending verbs. Specifically, the less likely events elicited larger negativity than highly likely events with short temporal shifts on the temporal indicator. On the sentence-ending word, the long temporal shift elicited enlarged negativity than the short temporal shift when the antecedent was highly likely. These findings have two key implications regarding the interplay of implied causality and falsity constraints during counterfactual comprehension. First, salient falsity constraints can override effects of causal coherence on processing. Second, greater negativity for unlikely antecedents suggests that counterfactual markers concurrently activate factual and hypothetical representations.

## 1. Introduction

Let us consider a real-world scenario: Wang signed up for the school activities. He prepared carefully. Readers may automatically infer an implied causal relationship between these temporally adjacent events (i.e., Wang needs to prepare carefully because he signed up). However, extending the temporal gap between events even minimally (e.g., to an hour) could weaken the perceived causality. Additionally, altering the likelihood of the antecedent event (e.g., Wang signs up for Arctic research vs. school activities) may change the causal strength. However, if we put this scenario into a counterfactual world (e.g., If Wang had signed up for the school activities, he would prepare carefully), how could the lengthening of temporal shift and the likelihood of the antecedent affect sentence comprehension?

Counterfactual conditionals are hypothetical statements structured as “If X had happened, Y would have happened”, allowing inferences about reality by considering alternatives that did not actually occur. For example, “If Wang had signed up for school activities, he would have prepared carefully” implies a causal relationship between Wang’s action and his preparation [1,2,3]. Two key constraints influence the comprehension of counterfactual conditionals. First, antecedent falsity refers to the antecedent describing a scenario known to be untrue, while the consequent suggests its consequences. Second, counterfactuals involve the perceived causality between the antecedent and consequent events [4], similar to causality in declarative statements [1,4,5,6].

However, it remains unclear how these constraints jointly impact counterfactual processing—an issue largely overlooked in the literature on complex sentence comprehension. Specifically, when causal coherence and antecedent falsity conflict, does one override or take priority over the other? This study reviews mixed evidence regarding the relative dominance of these counterfactual constraints. We selected two critical factors that modulate constraint strength—temporal shift length and antecedent likelihood—to probe their interaction during comprehension. Investigating how manipulating these variables influences processing can elucidate the underlying interplay governing counterfactual meaning construction. Determining the relative impacts of plausibility and implied causality holds important implications for gaining a broader insight into the mechanics of counterfactual language understanding. Clarifying how comprehenders negotiate conflicting semantic factors to build meaning advances theories examining the link between language and thought.

### 1.1. The Antecedent Falsity Constraints of Counterfactuals

A defining feature of counterfactuals is that the event described in the antecedent contradicts reality, strongly confirming antecedent falsity [1,5]. We conduct a literature review and the research shows two factors impacting falsity constraints. First, lexical devices like temporal indicators, negation, and conjunctions modulate counterfactual processing. Linguistically, temporal indicators are considered most critical [7,8,9]. Although there is limited psychological evidence to investigate how temporal indicators impact the antecedent falsity of counterfactuals, previous research of real-world scenarios showed that the effect of temporal indicators is primarily through extending or shortening temporal shifts. Extending temporal shifts between events in the antecedent could introduce additional processing costs [10,11,12,13]. These studies consistently demonstrated that longer temporal gaps could make it more difficult to access and integrate information about the prior event when processing the subsequent event. For example, Lucy petted the cat. After one second/hour/year, the cat purred happily. While research has examined such temporal distance effects in declaratives, how varying antecedent-consequent gaps impact counterfactual comprehension remains unknown. Previous psycholinguistics evidence showed that counterfactuals and real-world conditionals elicit similar neural responses to semantic anomalies (e.g., If dogs had gills/Because dogs have gills, Dobermans would breathe under water/poison) [14,15,16]. If counterfactuals pattern like real-world scenarios, longer temporal gaps between the antecedent and consequent may make it harder for readers to integrate information when encountering the consequent clause. On the contrary, others argue unrealistic counterfactual antecedents may more readily activate hypothetical representations [17,18], predicting reduced difficulty after long shifts compared to real-world scenarios.

The other influence on antecedent falsity is contextual information surrounding the antecedent clause itself. For example, research has examined whether transparent context establishing counterfactuality versus nontransparent context impacts processing (Dai, H., et al. [19]). Related studies investigate if logically consistent versus inconsistent context with the antecedent scenario alters effects (Haigh, M. and Stewart, A.J. [20], Stewart, A.J., et al. [21]). Specifically, Dai, H., et al. [19] compared the transparent counterfactual context (e.g., If everything in the world could go back in time…) to the nontransparent context (e.g., If better preparations were made at that time…) of the antecedent. They observed an N400 effect reflecting the consistency of a critical subsequent word with the prior context only for nontransparent counterfactuals (e.g., feel happy vs. sorry), while transparent conditions showed a later P600 effect. These findings demonstrate that antecedent transparency can modulate implicit counterfactuality, with downstream effects detectable even in the following clauses. 

Apart from context transparency, antecedent likelihood also impacts counterfactual processing. For example, Ferguson, H.J. and Jayes, L.T. [22] used eye-tracking while participants read counterfactuals with likely (Sophie using a knife) or unlikely (Sophie using a pump/axe) antecedents, followed by congruent consequents (preparing carrots for dinner). Reading times increased on the final word “carrots” for unlikely antecedents, indicating greater difficulty integrating improbable events with subsequent outcomes. Similarly, Ferguson, H.J. and Sanford, A.J. [18] compared counterfactuals (“If cats were vegetarian”) to real-world conditionals (“If cats are hungry”) combined with appropriate real-world (“feed them fish”) or counterfactual (“feed them carrots”) consequents. In the counterfactual “vegetarian” context, the mismatching “carrots” continuation showed increased first-pass reading time compared to the matching “fish” in the real-world scenario. This again suggests that comprehenders leverage background knowledge to evaluate antecedent plausibility, with unrealistic events disrupting subsequent integration [17]. 

### 1.2. The Implied Causal Relation between the Antecedent and the Consequent in Counterfactual Conditionals

While limited evidence examines factors influencing causal processing in counterfactuals specifically, insights can be drawn from related complex sentences conveying implicit causality. For instance, concession sentences with contrasting clauses also contain implied causal links, similar to counterfactuals. Several studies, e.g., [23,24], used the eye-tracking technique, self-paced reading, and the fMRI technique to examine the concessive sentences indicated by conjunction words (e.g., *jinguan* [although]) and causal conditionals with conjunction words (e.g., *yinwei* [because]). They also manipulated causal coherence by disrupting the implicit causal relationship between clauses, observing that both sentence types relied on overlapping neural regions important for reasoning processing, including Inferior Frontal Gyrus (IFG), Middle Frontal Gyrus (MFG)/Medial Prefrontal Cortex (mPFC), Posterior Middle Temporal Gyrus (pMTG), and Temporoparietal Junction (TPJ). However, concessive sentences elicited greater activation, indicating additional processing costs to infer implicitly conveyed causality. This implies that while concessive and causal conditionals both depend on constrained causal links between clauses, concessive relations require more effortful causal processing. Concessive sentences thus provide an informative analogy for understanding potential factors that may modulate implicit causal effects in counterfactual comprehension.

Compared to the conjunction words, previous theoretical research argues that the temporal indicator could be the most important lexical device to impact counterfactual processing [7,8]. However, how these devices impact the perceived causality between clauses remains unclear. For instance, studies show that reversing event order using “before” versus “after” increases N400 amplitude [25,26]. Readers must reorder events to restore the expected chronology, disrupting the original temporal and causal contingency. Relatedly, Liao, Q., et al. [9] delved into the temporal sequences (chronological vs. reverse chronological) by manipulating antecedent temporal indicators (e.g., *zuotian* [yesterday] vs. *mingtian* [tomorrow]) in Chinese counterfactuals. Their findings revealed that reverse chronological conditions elicited a significantly larger N400 and P600 amplitude compared to chronological conditions. Crucially, reversing order transforms causal direction from cause-to-effect into effect-to-cause, violating the expected temporal-causal bias and increasing processing demands. These findings suggest that manipulating implicit temporal relationships in counterfactuals may similarly attenuate causal links across clauses. Elucidating this interaction advances the understanding of factors modulating causal integration during comprehension.

Another key factor influencing causal strength in counterfactuals is the described likelihood of the antecedent event. Less likely events prompt stronger inferred causality about the consequent compared to more expected scenarios [27]. For instance, “If Chris had won the lottery, he would have quitted his job” implies a stronger causal connection than “If Chris had gotten a raise, he would have quitted his job” since winning the lottery has a lower probability. This effect extends to simple declarative sentences. Less expected events (e.g., “Charlie ate a big plate of veggies”) elicit stronger subsequent causal inferences than more expected equivalents (e.g., “Charlie ate a big plate of fries”) [28]. Critically, while temporal indicators influence antecedent falsity and causality similarly, antecedent likelihood has divergent effects—heightening causal connections but benefiting falsity processing for improbable events. Elucidating these competing impacts on comprehension can clarify the relative dominance of causal inference and falsity constraints when they conflict, advancing theories of counterfactual language understanding.

### 1.3. The Present Study

This study aimed to investigate whether and how the length of temporal shifts and the likelihood of the occurrence of the antecedent could change the processing difficulty of Chinese counterfactuals. By examining these factors, we can also elucidate whether counterfactual antecedents prompt dual factual-hypothetical representation activation versus solely factual activation, and explore causal and falsity constraint interaction.

Here, we conducted an ERP experiment with 2 (length of temporal shift: long vs. short) × 2 (the likelihood of the occurrence of the antecedent: less likely vs. highly likely) factorial design. Firstly, the operational definition of the temporal shift was the gap of temporal duration between two sequential events in the antecedent and the consequent (e.g., *qunian-xianzai* vs. *zuotian-xianzai* [last year-right now vs. yesterday-right now]). Since Mandarin Chinese lacks dedicated morphosyntactic markers for counterfactuality, Chinese speakers often rely on temporal indicators to convey counterfactual meaning. Specifically, past temporal indicators (e.g., *zuotian* [yesterday], *shangzhou* [last week]) can readily encode events as a counter to reality in Chinese counterfactuals. By locating the event on a clear past timeline, these past temporal indicators facilitate establishing the counterfactual implication that the event did not actually occur. This makes past indicators an important lexical device for conveying counterfactuality in Mandarin [7,29,30,31]. To keep the critical item constant between conditions, we varied the past temporal indicators in the antecedent to indicate differential locations on the chronological timeline but kept the temporal indicator the same across conditions. Secondly, we manipulated the likelihood of the occurrence of the antecedent by varying the plausibility of the described event in the antecedent of the counterfactual statements (e.g., *canjia beiji kekao* vs. *canjia xiaoyuan huodong* [signed up for Arctic scientific research vs. signed up for school activities]). 

Previous studies on the impact of contextual cues on online counterfactual processing chose the word in the consequent as the critical word [9,17,28,32]. The temporal indicator in the consequent serves as a critical word on which a temporal dependency can be formed across clauses. In this place, readers could access the temporal information and interpret it in the preceding context to build the relationship of the events in the antecedent and consequent of counterfactuals [9]. On the temporal indicator, the effects of temporal shifts and the likelihood of the occurrence of the antecedent can both be predicted from their impacts on the antecedent falsity constraint and on the implicit causality constraint. For the effect of temporal shifts, one prediction was that longer temporal shifts between antecedent and consequent events are expected to make the antecedent scenario seem more counter to reality. Moreover, increasing the temporal distance may also weaken the perceived causal relationship between the events described across the two clauses in reality. This enhanced counterfactual implication was expected to facilitate the construction of falsity of the antecedent event, thereby strengthening the falsity constraints during comprehension compared to short temporal shifts [33]. The increased temporal shift predicts an enhanced N400 regardless of its impact on the antecedent falsity or on the implied causality account. For the effect of the antecedent likelihood of the occurrence, antecedent events with low likelihood could facilitate the processing of falsity constraints by making it easier to construct a hypothetical scenario that is known to be false, reducing the N400 in the less likely event; however, these events could make it easier to infer an implied causal relationship between the antecedent and consequent compared to highly likely events, increasing the N400 in the less likely event. If the antecedent falsity constraint prevails, less likely conditions may reduce the N400 by benefiting falsity construction despite the causal constraint. But if the causal relationship takes priority, less likely events may increase the N400 if comprehenders focus more on the increased causal relation rather than the heightened falsity. 

We also predict a downstream effect of temporal shift and the likelihood of the occurrence of the antecedent on the sentence-ending word. We expect that readers might initiate a sentence-level wrap-up unification on the sentence-ending words. Such a process could trigger an updating of the sentence-level event situation model [34]. Specifically, an enlarged negativity effect could exist since participants could not effectively relate the final verb (e.g., *renzhen zhunbei* [prepare carefully]) to the prior situation model (e.g., *zuotian canjia beiji kekao* [signed up for the Arctic scientific research yesterday]; Jiang et al., 2013). Measuring effects on the sentence-ending verb can reveal if, and how, the relative importance of the causal and antecedent falsity constraints shifts based on the strength of each constraint across the sentence. For example, early effects from less likely events could attenuate the final word once the counterfactual context is established. The effects on the sentence-ending word could reveal a sustained impact of the interplay of causal semantic reasoning and the processing of antecedent falsity constraints during counterfactual sentence comprehension.

## 2. Materials and Methods

### 2.1. Participants

Twenty-eight Mandarin Chinese undergraduate students were recruited (13 males, mean age = 21.32 ± 1.73 years) for the EEG study. The sample size was estimated using Gpower 3.1.9.2 [35], with an alpha level of 0.05. Based on the effect size (η² = 0.158) reported in the previous study for the N400 effect of temporal distance [10], at least 24 participants were required for the main effect of temporal distance. Given that the actual number of participants (N = 28) was more than this estimated value, statistical power was ensured. The power analysis showed that at least 15 participants were required for the main effect of the length of temporal shift to reach the power of 95%. Given that the actual number of the current study (N = 28) was larger than this estimation, statistical power was ensured. One participant was excluded from data analysis due to excessive artefacts (over 25% of trials were rejected). All participants were right-handed and reported normal visual acuity. None reported to experience any psychiatric or neurological illness. Before the experiment, all provided informed consent. This study was approved by the Ethics Committee of Tongji University.

### 2.2. Design and Material

One hundred and twenty quadruplets of two-clause sentences were developed as critical stimuli (see Table 1). Each sentence was a Chinese counterfactual containing an antecedent and a consequent. The connective *Yaobushi* (if it had not been for) was selected as the counterfactual marker according to linguistics arguments [8,9,30,36], given that this word provided a strong counterfactual meaning based on behavioral judgments among related discourse markers [37]. We kept the consequents the same across different conditions, whereas differences existed only in the antecedent where the length of temporal shift and the likelihood of the occurrence of the antecedent were manipulated. The length of the temporal shift was determined by the temporal indicator in the antecedent and that in the consequent. The short (e.g., *zuotian-xianzai* [yesterday-now]) and the long (e.g., *qunian-xianzai* [last year-now]) length of the temporal shift were specified according to the consequence of altering the distance of the events in the antecedent and the consequent. The likelihood of the occurrence of the antecedent was defined as the plausibility of the events described in the antecedent. More specifically, the level of likelihood was classified into less likely (e.g., *xiaowang bao ming beiji kekao* [Wang has signed up for Arctic scientific research]) and highly likely (e.g., *xiaowang baoming xiaoyuan huodong* [Wang has signed up for school activities]). 

#### Validation Tests

Before EEG testing, five validation pretests were set up to obtain characteristics relevant to the experimental manipulations for the 140 sets of experimental sentence materials created. These tests aimed to validate the length of temporal shift, the likelihood of the occurrence of the antecedent event, the likelihood of the occurrence of what is described at sentence level, the strength of causal relation between the events in antecedents and consequents, and the sentence coherence. After removing 20 quadruplets with the lowest comprehensible scores, 120 sets were left for the other two pretests and EEG recordings. The descriptive measures of the three pretests were shown in Table 2. 

In the first pretest, sixteen native Chinese speakers who did not participate in the EEG study were required to evaluate the length of temporal shift between the events that occurred in the antecedent and consequent on a 7-point Likert scale (1 = extremely short, 7 = extremely long). The testing sentences were split into four lists, each given to four participants. The linear mixed effects model (LMEM) revealed that the main effect of the length of the temporal shift was significant, *F* (1, 15) = 70.51, *p* < 0.001, suggesting that readers could recognize the differences between the long (mean = 2.05) and short (mean = 4.98) temporal shift sentences. 

To evaluate the likelihood of the occurrence of the antecedent, another sixteen participants were recruited to estimate the likelihood of the description of the antecedent based on their real-world knowledge on a 7-point Likert scale (1 = highly unlikely, 7 = highly likely). The results showed that the main effect of the likelihood was significant, *F* (1, 15) = 16.42, *p* = 0.003. In particular, the score of the highly likely (mean = 6.22) conditions was higher than the highly unlikely (mean = 3.85) conditions. 

A third group of sixteen participants were invited to evaluate the likelihood of occurrence of what is described in the entire sentence on a 7-point Likert scale (1 = less likely, 7 = highly likely). The LMEM showed that only the effect of antecedent event likelihood was significant, *F* (1, 15) = 27.98, *p* < 0.001. Neither the main effect of the length of temporal shift (*F* (1,15) = 0.19, *p* = 0.66) nor the interaction effect of temporal shift and the likelihood of occurrence of antecedent events was significant, *F* (1, 360) = 0.08, *p* = 0.77, suggesting that the likelihood of the described antecedent events influenced perceptions of overall sentence plausibility, while the length of temporal shift did not impact judgments of sentence likelihood. 

A fourth group of 16 participants were invited to evaluate the strength of causality between the events in the antecedent and consequent on a 7-point Likert scale (1 = highly unacceptable, 7 = highly acceptable). The results clearly showed that the main effect of the antecedent likelihood was significant, *F* (1, 15) = 17.96, *p* < 0.001. The less likely conditions produced a higher score than the highly likely conditions. The main effect of the temporal shift was not significant, *F* (1, 15) = 10.15, *p* = 0.09. The interaction effect of temporal shift and likelihood of occurrence was significant, *F* (1, 671) = 17.32, *p* < 0.001. The follow-up analysis showed that the mean score of the short temporal shift in highly likely conditions (M = 4.65) was higher than the long temporal shift in highly likely conditions (M = 3.36), *β* = 0.60, *SE* = 0.14, *z* = 4.30, *p* < 0.001, 95% CI: [0.33, 0.87]. 

The critical manipulations could influence coherence ratings, which could further confound the interpretation of the behavioral and ERP results. To ensure that the participants’ evaluations of sentence coherence were not inadvertently impacted by temporal shift or antecedent likelihood, a last group of 16 new participants who did not participate in other pretests or EEG testing assessed the overall coherence based on whether these sentences were grammatically correct, semantically, or pragmatically comprehensible on a 7-point rating scale (1 = least acceptable, 7 = most acceptable). The LMEMs demonstrated that the main effect of the length of temporal shift (*F* (1, 15) = 2.28, *p* = 0.61), the likelihood of the occurrence of the antecedent (*F* (1, 15) = 0.11, *p* = 0.74), and the likelihood of the occurrence of the antecedent × the length of the temporal shift was all insignificant, *F* (1, 671) = 4.50, *p* = 0.30. 

### 2.3. Procedure

**Figure 1:**
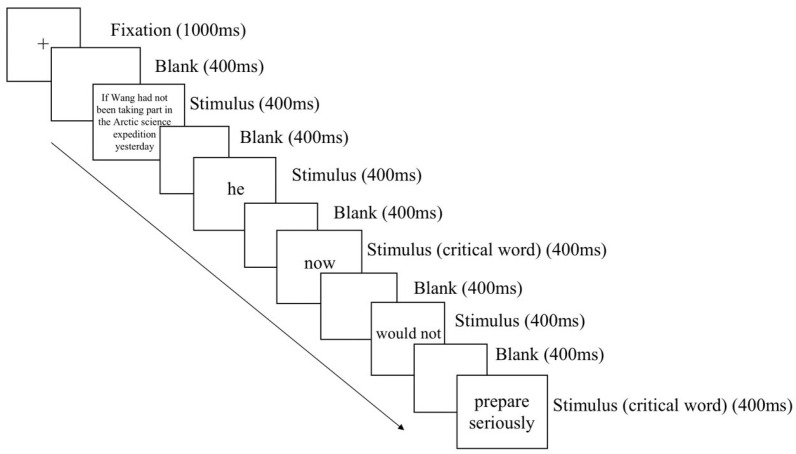
Stimulus presentation sequence in the present experiment.

The participants were seated in a dimly lighted and sound-attenuated room where all stimuli were presented on a computer monitor (32 inches in size, 1920 × 1080 in resolution, and 120 Hz in refreshing rate). Each trial began with a fixation cross at the center of the display for 1000 ms [38]. After a 400 ms blank screen, each sentence was visually presented word-by-word with a single word duration of 400 ms followed by a blank screen of a 400 ms interval (Figure 1). Participants were asked to press A or L on the keyboard to respond to a yes/no comprehension question. Experiments were programmed in E-Prime (Version 3.0; Psychology Software Tools, Pittsburgh, PA, USA). The aim of the questions was to test the participants’ comprehension of the sentences. The distribution of left/right hand to yes/no responses was counterbalanced across participants. Half of both the critical and filler trials were followed by comprehension questions. The number of questions with “yes” and “no” responses were balanced. At the beginning of the experiment, the participants received 10 practice trials to familiarize themselves with the procedure. The stimuli were separated into three blocks, with a three-minute break between each block. Including the time for setting up electrodes and completing practices, the whole experiment lasted for about 1 h per participant.

### 2.4. EEG Recording and Data Analysis

EEG data were collected from 32 channels mounted on an elastic cap following the standard 10–20 international system (Brain Products, Munich, Germany). The bio-signal was amplified and sampled at 500 Hz [39]. The EEGs were referenced online to FCz and offline to the mean of the left and right mastoids (TP9 and TP10) [29]. During the recording, the electrode impedance was kept below 5 kΩ [40]. Brain Vision Analyzer 2.0 was used to pre-process the EEG data. Continuous EEGs were first band-pass filtered between 0.3 and 40 Hz [41]. Ocular artifacts were rectified using the independent component analysis (ICA) method [42]. The EEGs were epoched from 200 ms before and 800 ms after the onset of the critical word and the sentence-ending word (Figure 1). A baseline correction was then conducted on the mean amplitude of 200 ms ERP before the target onsets. All epochs with EEG voltages exceeding ±50 μV were excluded from the subsequent data analysis [43], leaving 88.7% of the overall trials (23, 25, 24, and 28 trials for less likely short shift, less likely long shift, highly likely short shift, and highly likely long shift, respectively).

We selected two time windows to calculate the mean amplitudes per condition based on the literature on counterfactual sentence processing: 300–500 ms for the N400 (e.g., [19,44]), and 500–800 ms for late positivity (e.g., [45]). Mean ERP amplitudes per participant per condition were fitted with linear mixed effects models (LMEM). The models were built on the midline and lateral electrodes independently. The topographic factor for the midline analysis was electrode (Fz, Cz, and Pz). For the lateral analysis, the topographic factors contained hemisphere (left vs. right) and region (anterior vs. posterior). Four regions of interest (ROI) were formed with five representative electrodes per ROI across hemisphere and region: left anterior (Fp1, F7, F3, FC5, and FC1), left posterior (CP5, CP1, P7, P3, and O1), right anterior (Fp2, F8, F4, FC6, and FC2) and right posterior (CP6, Cp2, P8, P4, and O2). The amplitudes of each electrode were averaged before entering analysis for each ROI. The length of temporal shift (long vs. short), the likelihood of the occurrence of the event in the antecedent (highly likely vs. less likely), and topographic factors were treated as fixed factors. To evaluate the individual adjustments in the magnitude of ERP responses as a function of fixed factors, subjects were chosen as random intercepts [46,47]. Based on a model-selection procedure used by the likelihood ratio test [48], the fixed effects of the length of temporal shift, likelihood of occurrence, hemisphere, region, and their interactions for the lateral analysis, and length of temporal shift, likelihood of occurrence, electrode, and their interactions for the midline analysis, were included in the best-fitting model for analyzing ERP results. The *lme4* [49] and *lmerTest* packages [50] of R-studio (Version 3.1.0, http://cran.r-project.org (accessed on 6 May 2023) were used for all statistical analyses.

## 3. Results

### 3.1. Behaviors

Overall 83.7% of the comprehension questions following critical stimuli were answered correctly, suggesting that readers in general accurately responded to critical sentences. The binary response (correct vs. incorrect) was fit with the logistics mixed-effect model [51]. The model revealed that the main effect of the likelihood of the occurrence of the antecedent was significant, *β* = −0.42, *SE* = 0.14, *z* = −3.03, *p* = 0.002, 95% CI: [−0.69, −0.14], with a higher accuracy for likely (87.81%) versus unlikely (82.80%) sentences. The length of temporal shift x the likelihood of the occurrence of the antecedent interaction was also significant, *β* = −0.42, *SE* = 0.14, *z* = −3.03, *p* = 0.002, 95% CI: [−0.69, −0.14]. The follow-up analysis showed that the proportion of correctly judged responses was higher in the highly likely, short (91.10%) than in the less likely, short (79.13%) sentences. The analysis of reaction time did not show any main effect of the length of temporal shift (*F* (1, 26) = 0.65, *p* = 0.42, η^2^ < 0.001), the likelihood of the occurrence of the antecedent (*F* (1, 26) = 1.48, *p* = 0.24, η^2^ = 0.06), or the interaction between the two (*F* (1, 1484) = 1.24, *p* = 0.27, η^2^ < 0.001). 

### 3.2. ERPs

**Figure 2:**
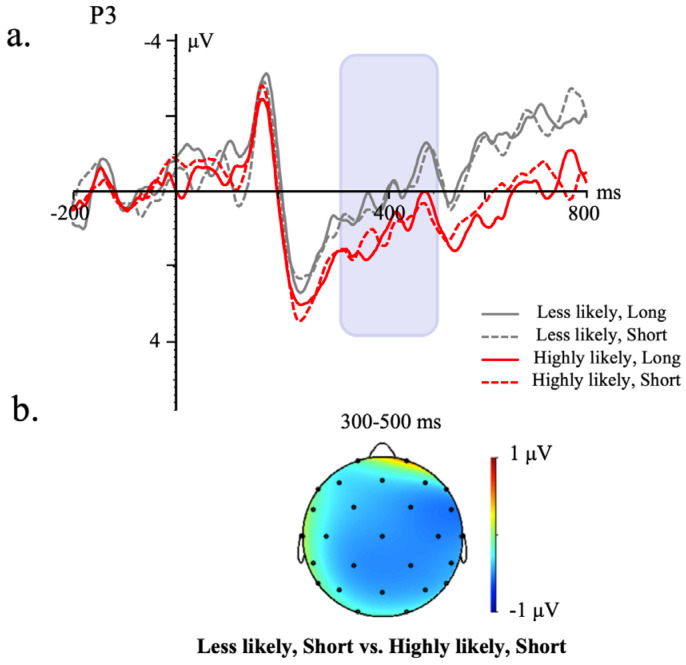
Grand average ERP waveforms time-locked to the onset of the temporal indicator of the consequent from −200 to 800 ms at a representative electrode. (**a**) represents the grand average waveform for all four conditions during the time window of −200 to 800 ms on a representative electrode. (**b**) illustrates the topographic maps of the mean amplitude difference between the “Less likely, short” and “Highly likely, short” conditions in corresponding time windows.

**Figure 3:**
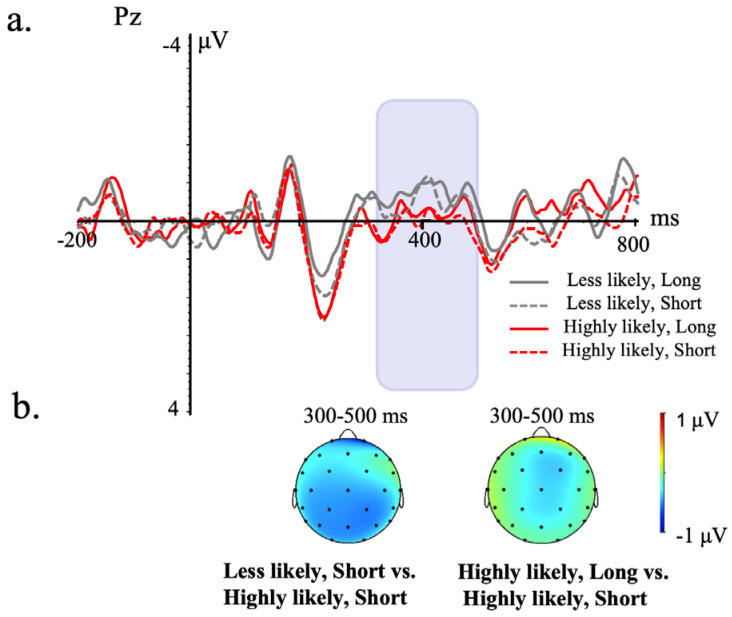
Grand average ERP waveforms time−locked to the onset of the sentence−ending word of the consequent from −200 to 800 ms at a representative electrode. (**a**) represents the grand average waveform for all four conditions during the time window of −200 to 800 ms on a representative electrode. (**b**) illustrates the topographic maps of the mean amplitude difference between the “Less likely, short” and “Highly likely, short” conditions in corresponding time windows.

Figure 2 and Figure 3 demonstrate the grand average ERP waveforms on a representative channel, time-locked to the temporal indicator and sentence-ending words of the 300–500 ms/500–800 ms time window. 

#### 3.2.1. Temporal Indicators of the Consequent

In the 300–500 ms time window, the lateral analysis showed that the likelihood of the occurrence of the antecedent significantly interacted with the length of temporal shift, *F* (1, 338) = 5.07, *p* = 0.03, η^2^ = 0.01. Follow-up analyses revealed that only in the short temporal shift condition, a larger N400 was shown in the less likely (−0.54 μV) than the highly likely condition (−0.17 μV) (*β* = −0.65, *SE* = 0.28, *t* = −2.36, *p* = 0.02, 95% CI: [−1.20, −0.11]), whereas no significant effect of the likelihood of the occurrence of the antecedent was found in the long temporal shift condition, *β* = 0.23, *SE* = 0.28, *t* = 0.83, *p* = 0.41, 95% CI: [−0.32, 0.78]. Neither the length of temporal shift (*F* (1, 26) = 0.80, *p* = 0.38, η^2^ = 0.04) nor the likelihood of the occurrence of the antecedent (*F* (1, 26) = 1,17, *p* = 0.28, η^2^ = 0.03) showed any significant main effect. No significant effect was found in the midline analysis (ps ≥ 0.06). 

In the 500−800 ms time window, no significant effect was shown in the midline or the lateral analysis (ps ≥ 0.07).

#### 3.2.2. Sentence–Ending Words

In the 300–500 ms time window, the midline analysis revealed a likelihood of the occurrence of the antecedent × the length of temporal shift interaction, *F* (1, 211) = 4.26, *p* = 0.04, η^2^ = 0.02. Follow-up analyses showed that the less likely condition (0.55 μV) elicited a greater negativity effect than the highly likely condition (1.36 μV) when the temporal shift was short, *β* = −0.90, *SE* = 0.40, *t* = −2.28, *p* = 0.02, 95% CI: [−1.70, −0.11]. Meantime, only in the highly likely condition, the long temporal shift (0.82 μV) elicited an enlarged negativity effect than the short temporal shift sentence (1.36 μV), *β* = −0.74, *SE* = 0.41, *t* = −1.78, *p* = 0.04, 95% CI: [−0.09, 1.56]. No significant effects were shown in the lateral analysis (ps ≥ 0.33). 

In the 500–800 ms window, no significant effects were shown in the lateral or the midline analysis (ps ≥ 0.25).

## 4. Discussion

The current study aimed to examine how temporal shifts of different lengths within the less likely and highly likely contexts impact counterfactual sentence comprehension. The ERP results showed that participants could immediately detect the likelihood of the occurrence of the antecedent based on their real-world knowledge in the short temporal shift conditions, enhancing the N400 effect on the temporal indicator of the consequent and negativity on the sentence-ending word. Moreover, when the antecedent events were highly likely, long temporal shifts elicited larger negativity than short temporal shift counterfactuals, particularly at the sentence-ending word. In the following paragraphs, we will discuss these findings and their related neurocognitive processes.

### 4.1. Temporal Shift and Counterfactual Processing 

While no neurolinguistic evidence exists examining temporal shifts in counterfactuals across likelihood contexts specifically, we can glean insights from related work. For example, Liao, Q., et al. [9] manipulated Chinese counterfactual antecedent temporal indicators to shift order from chronological (yesterday-tomorrow) to reverse (tomorrow-yesterday). Reversed sequences showed an enlarged N400 amplitude to consequent temporal words compared to chronological versions, attributed to the difficulty in reordering the mental timeline. However, the link between temporal sequence and implied causality was not thoroughly discussed. Crucially, reversing the sequence places effects before causes, violating the expected counterfactual causality. As Dancygier, B. [52] notes, temporal sequence intrinsically constrains causal direction in conditionals. For instance, in “The road was icy, and she slipped”, the earlier event implicitly takes causal precedence, despite both clauses potentially serving as the cause.

Similar to the constraint of temporal sequence to a preferred causal relation, the temporal shift also constrains the causal relation such that events with a close shift could be more causally appropriate. Past research on temporal shifts in non-counterfactuals has also shown a reduced negativity to words depicting events closely related in time, even when causal relationships are not strongly implied. For example, Ditman, T., et al. [10] manipulated temporal adverbial clauses by changing the temporal indicator between clauses (e.g., “after one second” vs. “after one hour”). They found that the negativity was systematically larger than the temporal word as the described time interval increased. This temporal shifting effect reflects an increased difficulty in extracting event information across longer time spans during comprehension [12,53]. It indicates a general processing cost of greater temporal distance irrespective of causal links. Counterfactuals typically imply a close temporal adjacency between the events in the antecedent and consequent, as seen in sentences like “If the road had not been so icy yesterday, she would not slip today” instead of “If the road had not been so icy last month, she would not slip today”. Shorter temporal shifts between clauses may confer additional benefits by supporting the construction of a more plausible causal relationship between the events. Some researchers propose that a close temporal proximity heuristically strengthens the perceived causality in conditional statements [54]. By this account, shorter shifts may aid causal linkage beyond just temporal sequencing in counterfactual processing. We should delineate these distinct facilitative factors of temporal proximity. Indeed, in the pretest of our study, we observed a similar trend in which highly likely counterfactual sentences with long temporal shifts were more demanding in establishing the causal relationship between the antecedent and consequent. When we increased the temporal shift between two events (e.g., “If the road had not been so icy last month, she would not slip right now”), the causal link between the antecedent and consequent weakened compared to short temporal shift conditions. Consequently, constructing the counterfactual meaning became more challenging. The observed negativity effect on the sentence-ending word could be attributed to the top-down retrieval-integration mechanism at a sentence/global level [34,55,56]. In highly likely conditions, the short temporal shifts could help readers use pre-activated semantic features to facilitate the integration of the events of the antecedent and consequent. On the contrary, when participants read longer temporal shift sentences, they could encounter a larger cost of semantic retrieval of the event in the antecedent to build an implied causal relation than in short temporal shift conditions [34,54,56], thus making the lexical representations in an event output layer more demanding. 

Our results were not consistent with Liao, Q., et al. [9]’s findings in which the temporal shift was also manipulated. In that study, the antecedent described an event which had not happened but was assumed to have occurred (e.g., “*yaobushi xiaowang zuotian meijiao lunwen…* [If Wang had handed in his paper yesterday]”). The temporal sequence of antecedent and consequent was also manipulated. Their results did not reveal significant differences in the 300–500 ms concerning long vs. short temporal shifts. This absence of significant N400 differences could potentially be attributed to two factors. Firstly, participants might prioritize the processing of the temporal sequence rather than focusing on the temporal shift, given that the temporal sequence could exert a more pronounced influence on the perception of causal relations, reducing the saliency of the temporal sequence. Secondly, our study is distinct from theirs since we focused on scenarios which contained the event that had actually existed but was assumed not to have been present (e.g., “*yaobushi xiaowang zuotian baomingle xiaoyuanhuodong*” [If Wang had not signed up for the school activity yesterday]). Hypothesizing a real event not to have occurred could demand a greater effort in negating what is described in the antecedent, making it more challenging for participants to construct mental representations of hypothetical events [17,57]. Consequently, this increased demand could hinder their ability to perceive differences in temporal shifts.

### 4.2. The N400 and Multiple Semantic Processes 

The manipulation of the likelihood of antecedent occurrences aims to elucidate how such likelihood may modulate the interplay between the implied causality and antecedent falsity constraints. One hypothesis posits that if participants prioritize the influence of implied causality over antecedent falsity constraint, the less likely events would lead to a decreased negativity compared to the highly likely events. Indeed, the pretest evaluating the strength of the causal relationship between the antecedent and consequent showed that the less likely sentences exhibited a stronger causal link than the highly likely sentences. This attenuation can be attributed to the functional priority of the causality constraint, despite the coexisting influence of the falsity constraint. However, contrary to this prediction, the current ERP results indicate a larger negativity elicited by less likely conditions specifically on the temporal indicator of the consequent in the context of short temporal shift conditions. This finding suggests that the impact of antecedent likelihood on falsity constraints could supersede its effect on causality across clauses.

Several factors may account for the observed effect of likelihood on antecedent falsity. Firstly, the causal relations in this study are less explicit and span across clauses. In contrast to the N400 effect observed in the current study, related research on other discourse markers (e.g., *lian…dou…*/Even) found that less likely events were easier to comprehend rather than highly likely events, reflecting an increased N400 followed by a late negativity or an increased bilateral IFG activation in the highly vs. less likely event [58,59]. However, the marker in this study functions to reverse the expectation of a less likely event in a single sentence rather than between clauses. Secondly, in the less likely condition, the antecedent event only challenged a reader’s stereotypical norm but did not violate the world knowledge (e.g., “sign up for school activities vs. Arctic research”). Xu, X., et al. [60] examined the concessive structure which also implied a causal relation of events between clauses. They manipulated the possibility of the events to form a causal relation or not (e.g., in “Grandma has moved from Harbin to Hainan, although she liked the winter there being warm and comfortable”, it is not possible for someone to like the warm winter in a place but not moving from a cold place to a warm place), and showed no N400 difference between possible and impossible conditions. Thirdly, the strength of causality could be attenuated in the hypothetical representation activated by *yaobushi* (If it had not been) compared with the statement. The pattern of our study is similar to relevant research on negation. In “A robin is not a tree/bird”, previous studies observed that *tree* evoked a larger N400 than *bird*, suggesting that the computation of the truth value based on the negation marker could fail when another semantic constraint was sufficiently strong (“A robin is a tree”) [61,62]. 

### 4.3. Limitations and Future Study

This study acknowledges certain limitations that warrant further consideration. Firstly, while the increased N400 for less likely antecedents indicates the activation of real-world representations, it remains debatable whether counterfactual antecedents prompt the dual activation of factual and hypothetical representations versus solely hypothetical representation without factual activation. The finding that less likely conditions elicited greater negativity could also result from a weaker counterfactual marker or a less salient antecedent violation. The less likely condition in our study may have a weaker sense of impossibility, potentially introducing a confounding bias. Future research should further explore whether this increased negativity could emerge even with weaker counterfactual markers, which would trigger signal factual representation during counterfactual processing. Secondly, although studies [7,37,63] have shown that the counterfactual marker used in the present experiment (*yaobushi…jiu* [If it had not been for, it would…]) conveys a meaning of strong deviation from the actual occurrence of the specified event, our study exclusively employed one counterfactual structure. Chinese theoretical linguists have argued that sentences with *yaobushi* (*if it had not been for*) are almost exclusively counted as counterfactuals, as these sentences are theoretically ranked bottom in the level of factuality and top in the level of hypotheticality [30]. Previous behavioral testing and corpus studies have shown that *yaobushi* has been regarded as a reliable predictor of counterfactuals (Hsu, 2014; Jing, 2017; Dai et al., 2019). In particular, Dai (2019) conducted a factuality rating (to ask readers to judge the extent to which the events framed by different conjunctions are interpreted as counterfactual) in sentences led by *yinwei* (if it had not been for), *ruguo* (in case of), and *yaobushi* (if it had not been for) and found only *yaobushi* conditionals were judged closest to the counterfactual type [19,31,63]. Chinese counterfactuals utilize diverse linguistic devices (e.g., *zaozhidao* [If I had known earlier], *ruguo* [If only]), generating a spectrum of counterfactuality that varies in the degree of such deviation. An intriguing avenue for future research lies in further exploring the interplay between multiple constraints in the counterfactual conditionals with the counterfactual markers varying in the strength of counterfactuality. Moreover, it is worth noting that various types of conditional sentences could share a common foundation of causal relationships that play a significant role in their comprehension. For instance, concessive conditionals and counterfactuals both involve causal relationships. Counterfactuals and concessives both involve negating reality, but use different linguistic forms. Counterfactuals create hypothetical scenarios that deny facts, through devices like past tense and subjunctives (e.g., “If it had rained…”). In contrast, concessives overtly contradict expected outcomes using connectors like “although” (e.g., “Although it rained…”). Therefore, the distinction between the hypothetical negation of counterfactuals and the overt denial of concessives could provide a deeper insight into their unique and shared semantic properties, and deserves further examination. Moreover, the interplay between antecedent falsity and implied causality observed in this study can inform future research that probes how multiple semantic constraints interact during the processing mechanisms of conditional reasoning in a broader sense. Additionally, the future direction of counterfactual processing research can further investigate connections with ongoing machine learning efforts to computationally model hypothetical thinking. For instance, Yanaka et al. [64] compared neural network models against human reasoning patterns over counterfactual utterances. Their work demonstrates the current capabilities but also the limitations of deep learning for context-dependent counterfactual analysis relative to human comprehenders. Meanwhile, Lewis and Steedman [65] proposed an integrated model combining statistical representations of word meanings with logical formalisms to compositionally determine counterfactual semantics. Findings from such computational studies elucidate complementary insights on counterfactual language understanding beyond behavioral experiments alone. Going forward, future research could adopt interdisciplinary methods joining empirical results with computational modelling techniques to enable more ecologically valid theories of counterfactual cognition grounded in the intricacies of real-world reasoning. 

## 5. Conclusions

The current study explored the influence of temporal shift length and antecedent likelihood on the comprehension of Chinese counterfactuals. The findings indicated two significant outcomes. Firstly, the impact of causal relationships could be overridden by salient antecedent falsity, suggesting an interplay between the processing of implied causality and antecedent falsity during counterfactual comprehension. Secondly, the increased negativity for less likely antecedents demonstrates that counterfactual markers could activate factual representations. This observation provides empirical evidence that comprehenders concurrently activate both factual and hypothetical representations when processing counterfactuals. Additionally, our study significantly contributes to the understanding of general counterfactual processing. Specifically, we shed light on the influential factors related to counterfactual premises, exploring the dynamic relationship between lexical semantics and logical semantics. By delving into the intricacies of the relationship between antecedent and consequent elements, we provide valuable insights into how these components interact in the processing of counterfactual statements. Furthermore, our research extends its impact on the processing of discourse markers and conditions, revealing their nuanced roles in counterfactual processing.

## Figures and Tables

**Table 1:** Sentence exemplars in one set of experimental stimuli.

Condition	Sentence Exemplar
Less likely, short	*yaobushi*/Wang/yesterday/signed up for Arctic scientific research, he/now/would not/prepare seriously/ If Wang had not been taking part in the Arctic science expedition yesterday, he would not have prepared seriously now.
Less likely, long	*yaobushi*/Wang/last year/signed up for Arctic scientific research, he/now/would not/prepare seriously/ If Wang had not been taking part in the Arctic science expedition last year, he would not have prepared seriously now.
Highly likely, short	*yaobushi*/Wang/yesterday/signed up for school activities, he/now/would not/prepare seriously/ If Wang had not been taking part in the school activities yesterday, he would not have prepared seriously now.
Highly likely, long	*yaobushi*/Wang/last year/signed up for school activities, he/now/would not/prepare seriously/ If Wang had not been taking part in the school activities yesterday, he would not have prepared seriously now.

Note: The critical words are underlined. Both literal and free translations are provided.

**Table 2:** Mean scores and standard deviations in the five pretests.

Condition	Length of Temporal Shift (1–7)		Likelihood of the Occurrence of the Antecedent Event		Strength of Causal Relationship		Likelihood of the Occurrence of What Is Described at Sentence Level (1–7)		Sentence Coherence (1–7)	
	M	SD	M	SD	M	SD	M	SD	M	SD
Less likely, short	2.21	1.29	3.81	1.28	5.88	1.79	3.38	1.96	6.13	1.20
Less likely, long	4.81	1.69	3.89	1.27	5.84	1.75	3.77	1.99	6.27	0.77
Highly likely, short	1.88	1.25	6.29	1.43	4.65	1.78	5.64	1.34	6.21	0.94
Highly likely, long	5.14	1.69	6.15	1.66	3.36	1.62	5.60	1.29	6.16	1.03

## Data Availability

The data presented in this study are available on request from the corresponding author. The data are not publicly available due to privacy.
